# Selective Q‑Band Excitation Modulates Exciton–Exciton Annihilation in Tetra(*p*‑Sulfonatophenyl)porphyrin J‑Aggregates

**DOI:** 10.1021/acsphyschemau.6c00064

**Published:** 2026-07-04

**Authors:** Maria Francesca Cherchi, Teresa Paviglianiti, Dario Baldon, Federico Toffoletti, Elisabetta Collini

**Affiliations:** Department of Chemical Sciences, 9308 University of Padova, Padova 35131, Italy

**Keywords:** porphyrins, J-aggregates, excitons, exciton−exciton annihilation, ultrafast dynamics, Q bands, 2D electronic spectroscopy

## Abstract

The ultrafast photophysics of the Q-band manifold of tetra­(*p*-sulfonatophenyl)­porphyrin (H_2_TPPS) is investigated by two-dimensional electronic spectroscopy (2DES) in both its monomeric and J-aggregated forms. By selectively exciting different regions of the Q bands, we uncover strongly excitation-dependent relaxation dynamics in the aggregates. Photoexcitation centered on the lower-energy Q transition leads to pronounced interband coupling and clear signatures of exciton–exciton annihilation, whereas selective excitation of the higher-energy side of the Q-band manifold limits annihilation. These findings provide new insight into how the mixed Q-band structure governs excitation flow in porphyrin aggregates and open avenues for the rational design of materials with tailored annihilation properties.

## Graphical abstract

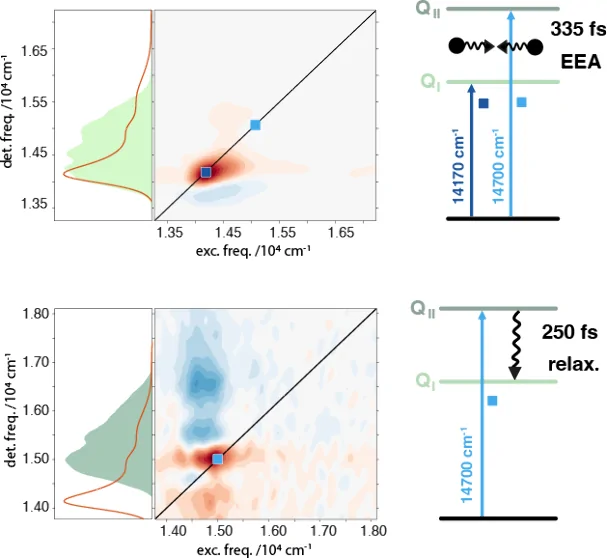

## 1 Introduction

Self-assembled porphyrin aggregates have long represented an archetypal molecular platform capable of mimicking natural photosynthetic architectures and providing essential insights into light-harvesting processes.^1–4^ These properties originate from the formation of delocalized photoexcited states—referred to as excitons^5^—which impart distinctive electronic characteristics and enhanced linear and nonlinear optical properties.^6^ Compared with individual monomers, self-assembled porphyrin derivatives exhibit unique collective phenomena including exciton coupling,^7^ photoinduced electron and charge transfer,^8^ as well as semiconducting and photoconducting capabilities.^9^ Consequently, beyond their biological fascination, the relevance of porphyrin-based assemblies extends to the design of multiphotofunctional materials, including those aimed for quantum coherent energy transport,^10^ and to the exploration of fundamental mechanisms of energy transport within extended π-conjugated frameworks.^11–13^ Additionally, owing to their high extinction coefficients in the visible and near-infrared (NIR) regions, corresponding to the maximum of the solar flux, they are considered promising candidates for solar light conversion purposes.^14,15^

To fully harness the directional control of energy flow within these porphyrin aggregates, it is essential to deepen our understanding of exciton–exciton interactions—specifically, how monomeric units communicate in response to external stimuli and how they collectively give rise to emergent exciton phenomena.

In recent decades, considerable attention has also been devoted to processes related to the spatial diffusion of excitons and their subsequent fate. Among these, exciton–exciton annihilation (EEA) is a key nonlinear phenomenon. In this process, two excitons interact such that one exciton relaxes to the ground state, while the other is promoted to a higher-lying excited state, which subsequently relaxes back to the one-exciton manifold, dissipating excess energy via phonon emission.^16,17^ EEA has been extensively studied in molecular aggregates,^18–21^ as it constitutes a loss channel limiting energy-transport efficiency, but it may also play a functional role in charge-separation processes.^22,23^ The identification and mitigation of EEA are thus crucial for the development of high-efficiency optoelectronic materials.

In this study, we investigate the ultrafast optical dynamics of the diacid form of the water-soluble tetra-(*p*-sulfonatophenyl)­porphyrin (H_2_TPPS) in both its monomeric and J-aggregated forms (Figure 1a). Within Gouterman’s four-orbital model,^24^ porphyrins exhibit high-energy B (Soret) transitions near 400 nm and lower-energy Q-band transitions spanning the 500–700 nm region. While the Soret-band dynamics of H_2_TPPS J-aggregates have been extensively characterized using two-dimensional electronic spectroscopy (2DES) and transient absorption,^25–28^ a comparable understanding of the Q-band manifold is still lacking. Unlike the B bands, Q bands display complex spectral features arising from symmetry breaking and strong electronic–vibrational coupling, making their properties highly sensitive to macrocycle substitution and environmental conditions. Yet, as the lowest-energy electronic states, Q bands play a central role in technological applications such as photovoltaics,^15,29^ optoelectronics,^30^ and photodynamic therapy,^31^ and they govern energy funneling in chlorophyll-based light-harvesting systems.^32^

**Figure 1:**
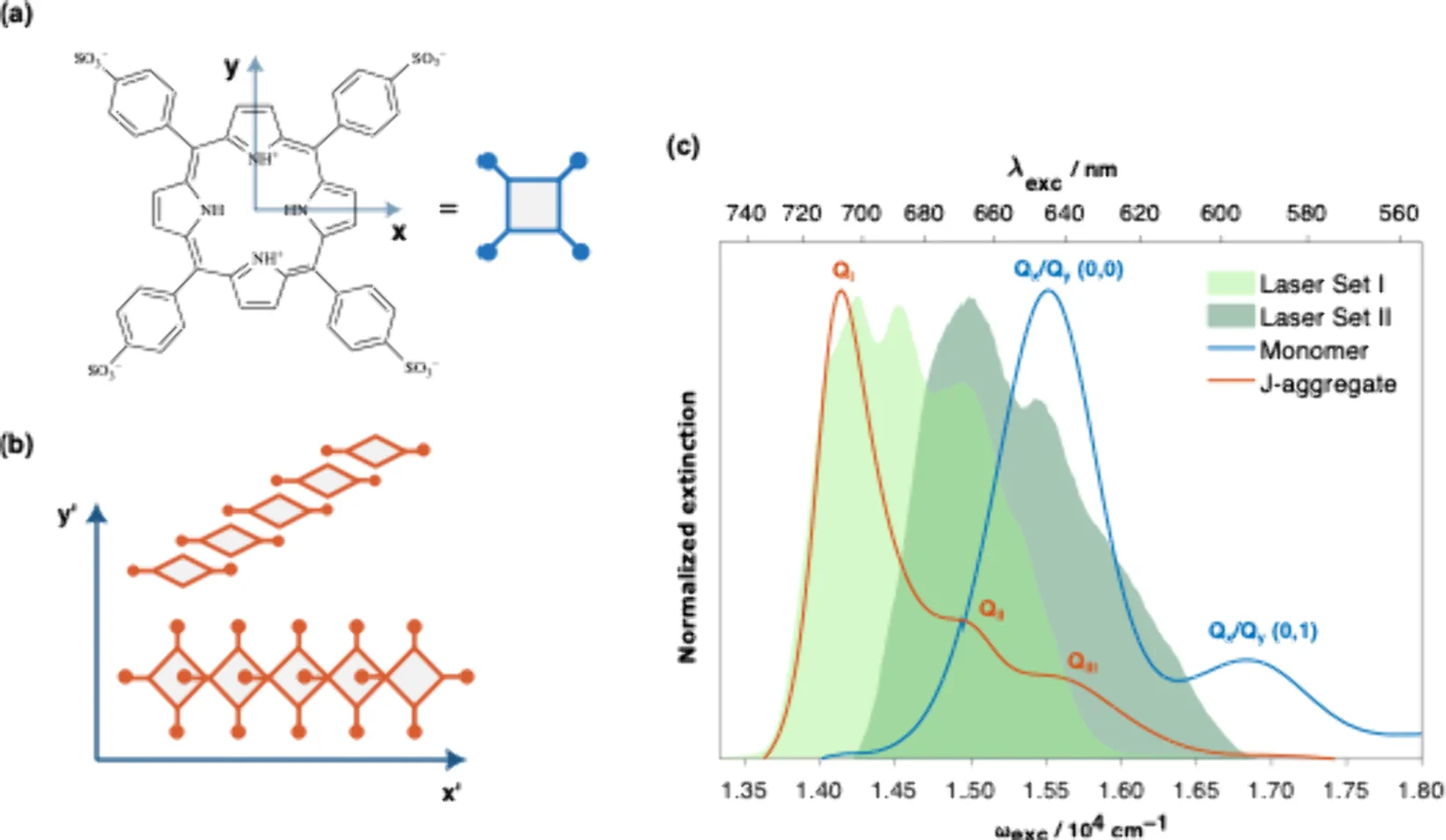
(a) Molecular structure of the diprotonated, water-soluble tetra-(*p*-sulfonatophenyl)­porphyrin (H_2_TPPS). The blue arrows indicate the orientation of the Q_*x*_ and Q_*y*_ transition dipole moments. (b) Schematic representation of the ideal collinear self-assembled geometry of the H_2_TPPS J-aggregate (top, side view; bottom, top view). The coordinate system was chosen such that the *y*′-axis is perpendicular to the line connecting the centers of the molecules. Interactions between the monomer transition dipole moments along the **x*′* and *y*′ directions give rise to the Q_I_, Q_II,_ and Q_III_ excitonic bands. The components of the transition dipole moments along the *x*′ axis are arranged to produce a J-type aggregation, whereas along the **y*′* axis they form an H-type arrangement (see ref 33 for further details). (c) Steady-state absorption spectra in the Q-band region of the H_2_TPPS monomer (blue) and J-aggregate (orange) in acidic aqueous solutions, overlaid with the excitation laser spectra used for the two 2DES measurement sets (Set I, light green; Set II, dark green).

Here, we present a comprehensive 2DES study of the Q-band dynamics of H_2_TPPS in both monomeric and J-aggregated forms. By selectively exciting different regions of the Q-band manifold, we uncover distinct excitation-dependent ultrafast relaxation pathways.

## 2 Materials and Methods

Tetra­(*p*-sulfonatophenyl)­porphyrin was obtained from PorphyChem and used without further purification. An acidic aqueous solution of H_2_TPPS (pH 2.2, 0.1 M HCl) was prepared for the monomer characterization, while the corresponding J-aggregate was obtained upon the addition of NaCl.

Multidimensional optical spectroscopic characterization was carried out using a fully noncollinear BOXCARS 2DES setup. Briefly, a Ti:sapphire Coherent Libra laser system (800 nm, 3 kHz, 0.8 mJ) coupled to a noncollinear optical parametric amplifier (NOPA) (Light Conversion TOPAS White) was used to generate the excitation pulses. Pulse compression to approximately 10 fs was achieved using a prism compressor in combination with a Fastlite Dazzler acousto-optic pulse shaper. The H_2_TPPS monomer was excited with a laser spectrum centered at 15260 cm^–1^ (655 nm). The H_2_TPPS J-aggregates were investigated under two excitation conditions: using the same spectral bandwidth as for the monomer measurements and using a spectrum centered at 14535 cm^–1^ (688 nm). In all experiments, the pulse energy at the sample position was maintained at 7–8 nJ per pulse. Further details on sample preparation, 2DES setup, and data analysis are provided in Sections S1 and S2 of the Supporting Information.

## 3 Results and Discussion

### 3.1 Linear Characterization and Electronic Properties

Linear optical characterization of the systems was performed in aqueous solutions (pH = 2.2), and the corresponding absorption spectra are shown in Figure 1b together with the laser emission profiles employed for the 2DES measurements. Details on sample solution preparation are provided in the Supporting Information.

In the spectral region of interest, the H_2_TPPS monomer features a pronounced band at 645 nm (15504 cm^–1^), attributed to the (almost) degenerate Q_*y*_ and Q_*x*_ electronic transitions, along with a weaker band at shorter wavelengths, 590 nm (16949 cm^–1^), assigned to a vibronic replica.^33–35^ Upon aggregation into the J-type configuration, an overall red shift and narrowing of the absorption features are observed.^33^ Three absorption bands are observed in the Q region, labeled in Figure 1b as Q_I_, Q_II_ and Q_III_, respectively. Their maxima are located at 706 nm (14160 cm^–1^), 667 nm (14990 cm^–1^) and 635 nm (15727 cm^–1^), in agreement with the well-known and extensively characterized behavior of H_2_TPPS J-aggregates.^26,36^ The origin of these new bands can be understood by considering that, upon aggregation, the degeneracy of *x-* and *y-*polarized transition dipole moments of the monomers is lifted. As a result, exciton formation leads to a splitting of the characteristic B and Q bands into red-shifted J- and blue-shifted H-bands (Bethe splitting).^37^ While the absorption profile in the B band region can be successfully modeled by considering excitonic couplings alone,^25,33,36^ an accurate description of the Q-band absorption requires explicit inclusion of coupling to molecular vibrations.

Theoretical predictions suggest that the lowest-energy absorption band (706 nm, Q_I_) originates predominantly from Q_*x*_ molecular transitions, whereas the second band (667 nm, Q_II_) is mainly Q_*y*_ in character.^33^ The highest-energy band (635 nm, Q_III_) arises from a mixture of the first vibronic replicas of Q_I_ and Q_II_ and includes contributions from multiple states with different polarizations and relatively small oscillator strengths.^33^

### 3.2 2DES Characterization of the H_2_TPPS Monomer

The 2DES measurements were performed using a fully noncolinear experimental setup as described in ref 38. Further details of the experimental conditions are provided in the Supporting Information.

The 2DES response of the monomer sample presents all the characteristics expected for monomeric tetrapyrrolic dyes in solution.^39,40^ A selection of absorptive 2DES maps at different *t*_2_ delays is summarized in Figure 2a, while additional rephasing and nonrephasing maps are reported in Figure S2 of the Supporting Information. At all population times *t*_2_, 2D maps are dominated by a positive diagonal peak centered around (15400, 15400) cm^–1^, originating from ground-state bleaching (GSB) and stimulated emission (SE) related to the main Q_*y*_/Q_*x*_ electronic transition. The signal appears elongated along the horizontal axis, due to the presence of off-diagonal features centered at coordinates of about (16500, 15400) and (17200, 15400) cm^–1^, which persist at all population times. This behavior is well documented for tetrapyrrolic chromophores in solution and is attributed to vibronic coupling and the contribution of higher vibronic states, which are also responsible for the vibronic progression observed in the linear absorption response.^39,40^ A qualitative inspection of the time evolution of the 2DES maps highlights that the initially elliptical shape of the positive peak gradually evolves into a more circular profile, reflecting the hallmark signature of spectral diffusion.^41^ This process is commonly observed in the relaxation dynamics of isolated organic dyes, where fluctuations of the surrounding environment induce variations in the electronic transition frequencies.^40,42–44^

**Figure 2:**
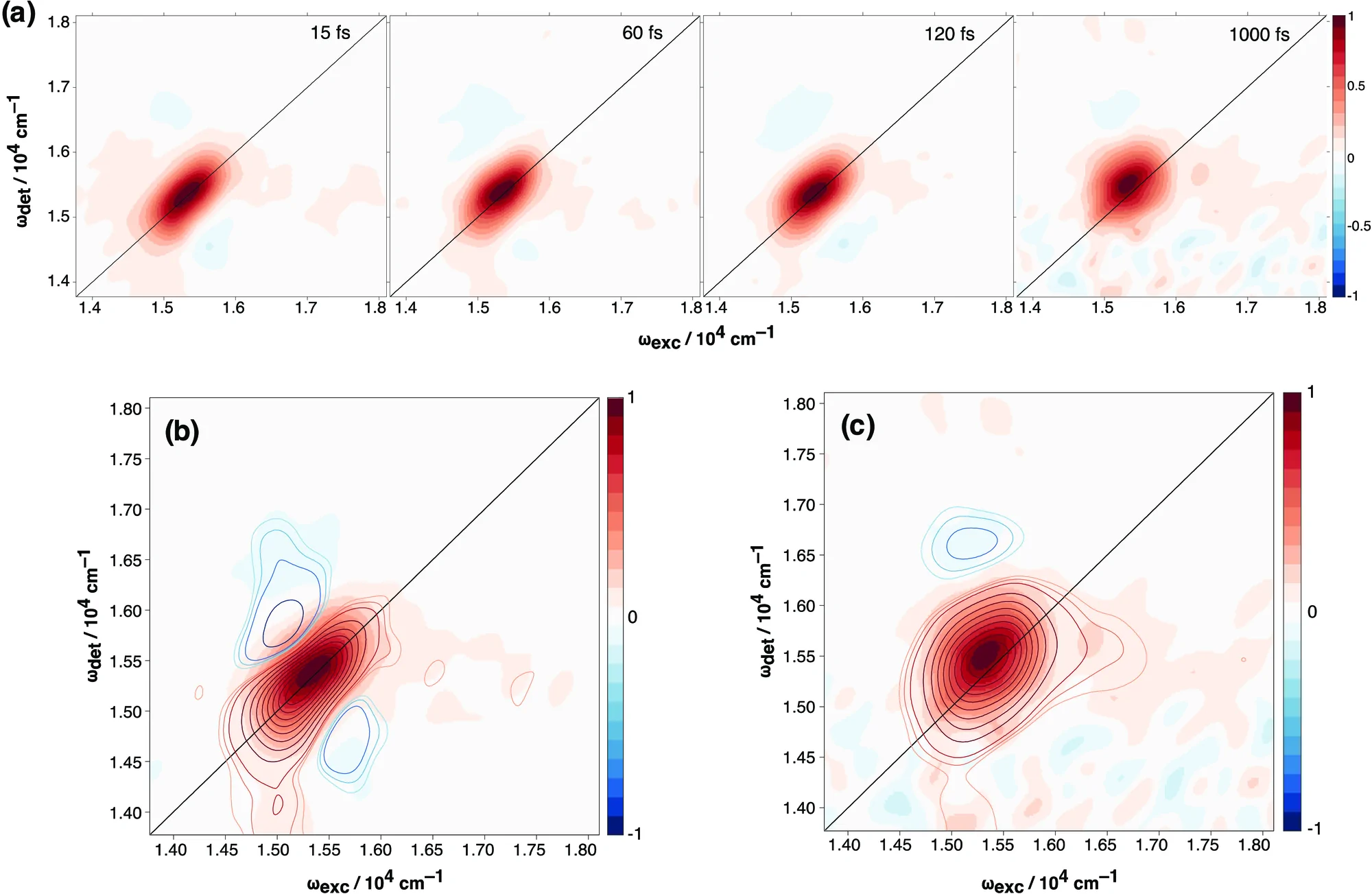
(a) Purely absorptive 2DES maps for the monomer sample at selected values of population time *t*_2_. (b) 2D-DAS map (contour) and corresponding absorptive 2DES map (background) associated with the 101 fs time constant. (c) 2D-DAS map (contour) and absorptive 2DES map (background) at the picosecond time scale. In both panels, the color scale is normalized to the 2DES map.

The excited-state dynamics of the system were better characterized using a global multiexponential analysis, which provided a comprehensive decomposition of the overall signal into decaying and oscillating components.^45^ The nonoscillating evolution of the transient signal can be described with a biexponential function with a fast time constant of 101 fs and a second, much longer component whose time constant exceeds the investigated time window (≫1 ps).

The amplitude distributions of these two kinetic components are reported in the 2D-decay associated spectra (2D-DAS) of Figure 2b,c, overlaid on the corresponding 2DES maps at comparable time scales. By examination of the sign and position of the signals in the 2D-DAS, valuable insights into the nature of the relaxation pathways and the states involved in the dynamics can be gained. In particular, for a positive signal in the 2DES map, a positive amplitude (red) in the 2D-DAS corresponds to a signal that decays with the associated time constant, whereas a negative amplitude (blue) indicates a signal that rises on that time scale.

In the 2D-DAS of the fast component (101 fs, Figure 2b), the concurrent decay of the signal along the diagonal and the rise of the signal in regions above and below it account for the rounding of the main diagonal positive feature, indicating a loss of excitation–emission frequency correlation, as expected during spectral diffusion. The 101 fs time constant is consistent with values commonly reported for the solvation dynamics and spectral diffusion in similar molecular dyes, such as chlorophylls.^39,42,46^

In contrast, the second 2D-DAS (Figure 2c), associated with a time constant exceeding the picosecond time scale, can be attributed to relaxation of the system back to the ground state, involving dynamics that occur on time scales much longer than those explored in the present experiment.

In addition to the decay dynamics, weak oscillations are observed in the signal evolution as a function of *t*_2_. Power-spectrum analysis was employed to preliminarily identify the frequencies of the dominant oscillatory components. To this end, the nonoscillatory contributions were removed from the total signal, and the resulting oscillatory residuals, integrated along both the excitation and detection axes, were Fourier-transformed with respect to *t*_2_.^47^ The resulting power spectrum (see Figure S6a of the Supporting Information) shows a pronounced peak at 241 cm^–1^, which, in agreement with Raman data reported in the literature,^48,49^ is assigned to an out-of-plane ruffling vibrational mode, known to be strongly coupled to the resonant Q electronic transition.^50^ This mode was also captured by the global fitting analysis, which allowed estimation of a dephasing time of approximately 500 fs and provided its amplitude distribution, visualized as coherence-associated spectra (2D-CAS), analogous to the 2D-DAS previously introduced for the nonoscillatory components. The 2D-CAS for this vibration, shown in the Supporting Information (Figure S7), exhibits the characteristic “chair-like” pattern predicted for vibrational beatings.^51^

Additional modes, barely emerging from the noise floor, were identified at 343, 434, 825, 888, 968, and 1334 cm^–1^, attributed to pyrrole vibrational modes, and at 640 cm^–1^, associated with a phenyl-ring vibrational mode.^52,53^ For these modes, the global fitting analysis could not yield further information regarding their damping times or amplitude distributions due to the low signal intensity. This behavior is typical of tetrapyrrolic chromophores, which exhibit low Huang–Rhys factors for their vibrational modes.^54,55^

### 3.3 2DES Characterization of the H_2_TPPS J-Aggregate

Figure 3 shows representative frames of the temporal evolution of the purely absorptive 2DES maps of the H_2_TPPS J-aggregates acquired under two different excitation conditions. Additional rephasing and nonrephasing maps are reported in the Supporting Information, Figures S3 and S4.

**Figure 3:**
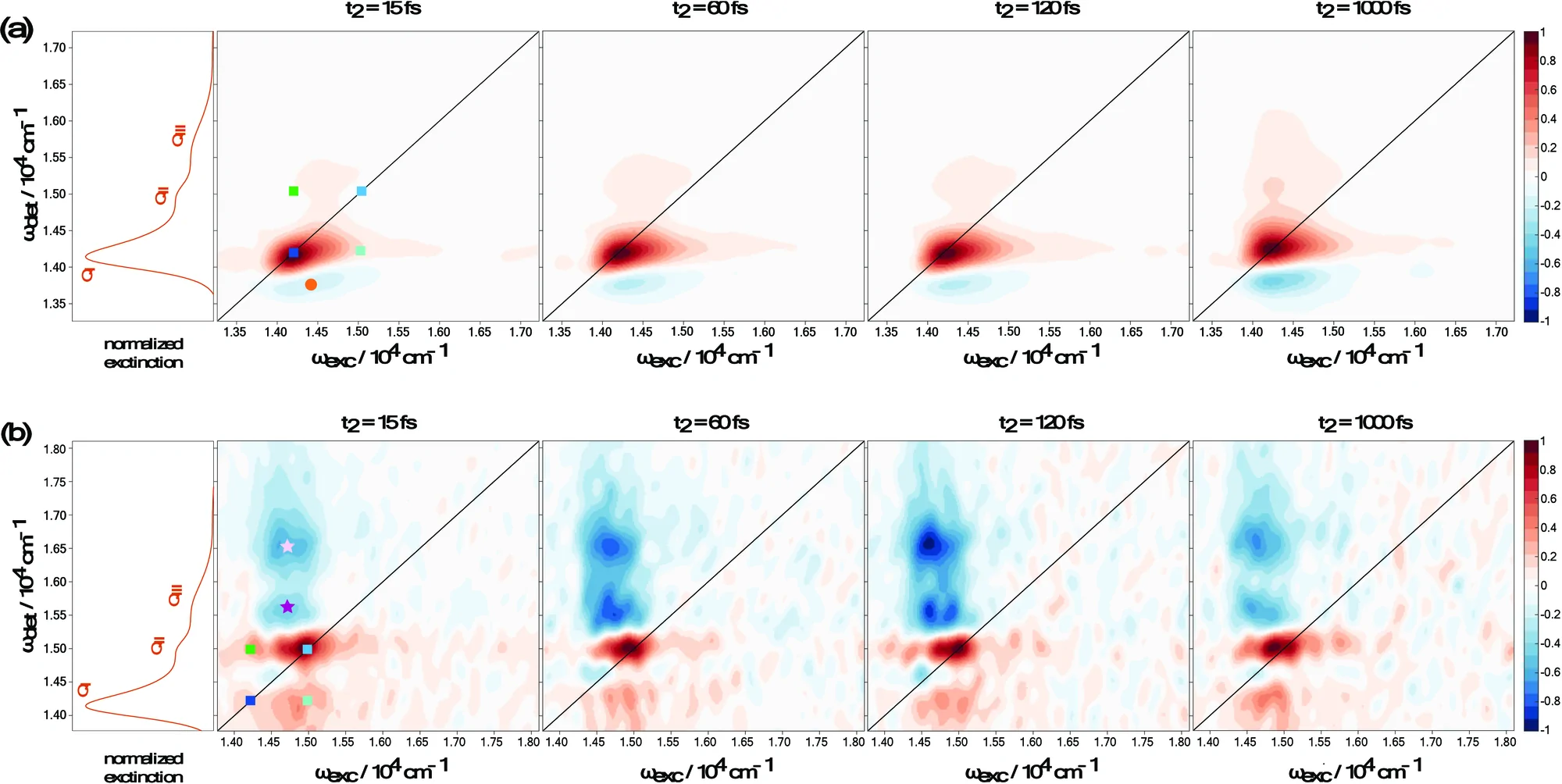
Purely absorptive 2DES maps of H_2_TPPS J-aggregate at selected values of population time *t*_2_. The maps were acquired using two distinct laser-pulse profiles: (a) Set I and (b) Set II. The pulse profiles employed in the two measurement sets are shown in the left panels (colored areas) overlaid with the linear absorption spectrum of the aggregates (orange line). Colored symbols pinpoint relevant coordinates discussed in the main text.

In the first set of measurements (set I, Figure 3a), the excitation bandwidth was designed to cover all Q-band transitions, with the aim of revealing potential signatures of interband coupling. In contrast, for the second pulse profile (set II, Figure 3b), the same spectral profile used for the monomer characterization was employed.

Under these conditions, excitation of the Q_I_ transition was excluded, thereby isolating dynamics associated exclusively with the higher-energy Q_II_ and Q_III_ bands.

The 2DES maps of Set I (Figure 3a) are dominated by a positive diagonal peak located at (14170, 14170) cm^–1^ (dark blue square marker), corresponding to GSB and SE pathways involving the Q_I_ band. A weaker diagonal feature at (15000, 15000) cm^–1^ (light blue square marker) is also observed and can be similarly attributed to the Q_II_ band. The reduced amplitude of this feature reflects both the lower oscillator strength of the Q_II_ band and the spectral profile of the excitation pulse.

Notably, off-diagonal peaks at cross coordinates between the two diagonal features (green and light-green square markers) are clearly visible. These cross-peaks appear at early population times immediately following photoexcitation and, together with the diagonal peaks, form the characteristic square pattern expected for strongly coupled systems in which the probed electronic transitions share a common ground state.^56,57^ This pattern persists throughout the entire measurement window, providing clear evidence of mixing between the Q_I_ and Q_II_ one-exciton bands. This finding is particularly significant in the context of aggregates as biomimetic light harvesters, as the origin and functional role of mixed Q-bands remain an open question in the complex energetic and spatial organization of photosynthetic antenna complexes and in the light-harvesting efficiency of green plants and algae.^58,59^

In addition to the positive features discussed above, a negative excited-state absorption (ESA) signal is detected from early population times at detection frequencies red-shifted relative to the main diagonal peak (orange circle marker). The excitation frequency coordinates of this feature span both the Q_I_ and Q_II_ bands, indicating that the ESA involves photoinduced absorption originating from both energy levels.

In ideal J-aggregates, selection rules predict that the transition from the ground state to the one-exciton manifold occurs at lower energy than the transition from the one- to the two-exciton manifold. Consequently, ESA features in pump–probe and 2DES spectra are expected to appear at probe (detection) frequencies blue-shifted with respect to the ground-state bleaching signal.^60–63^ This expectation is clearly not fulfilled in the present case. Similar red-shifted ESA features have been reported in previous pump–probe studies upon resonant excitation of the Q-band of H_2_TPPS J-aggregates.^27,28,50^ It was proposed that the observed ESA may involve a transition to a biexciton level, located energetically below the two-exciton manifold.^27^ Biexcitons are bound pairs of excitons originating from exciton–exciton interactions.^64,65^ Another possible interpretation is that the ESA accesses a partially dark state, reachable only through indirect excitation pathways, or a charge-transfer state, which have been previously proposed to be responsible for faster fluorescence decay lifetimes.^66,67^ 2DES measurements do not allow for the unambiguous determination of the nature of the final state reached by ESA. Additional targeted investigations, such as higher-order pump–probe and multidimensional spectroscopies, would therefore be required to clarify its origin.^68,69^

As for the H_2_TPPS monomer, also in this case, the dynamic evolution of the signals was evaluated by a global multiexponential fitting analysis. This analysis revealed three nonoscillatory damping constants: 30 fs, 335 fs, and a slower decay exceeding 1 ps. The associated 2D-DAS are reported in Figure 4a–c.

**Figure 4:**
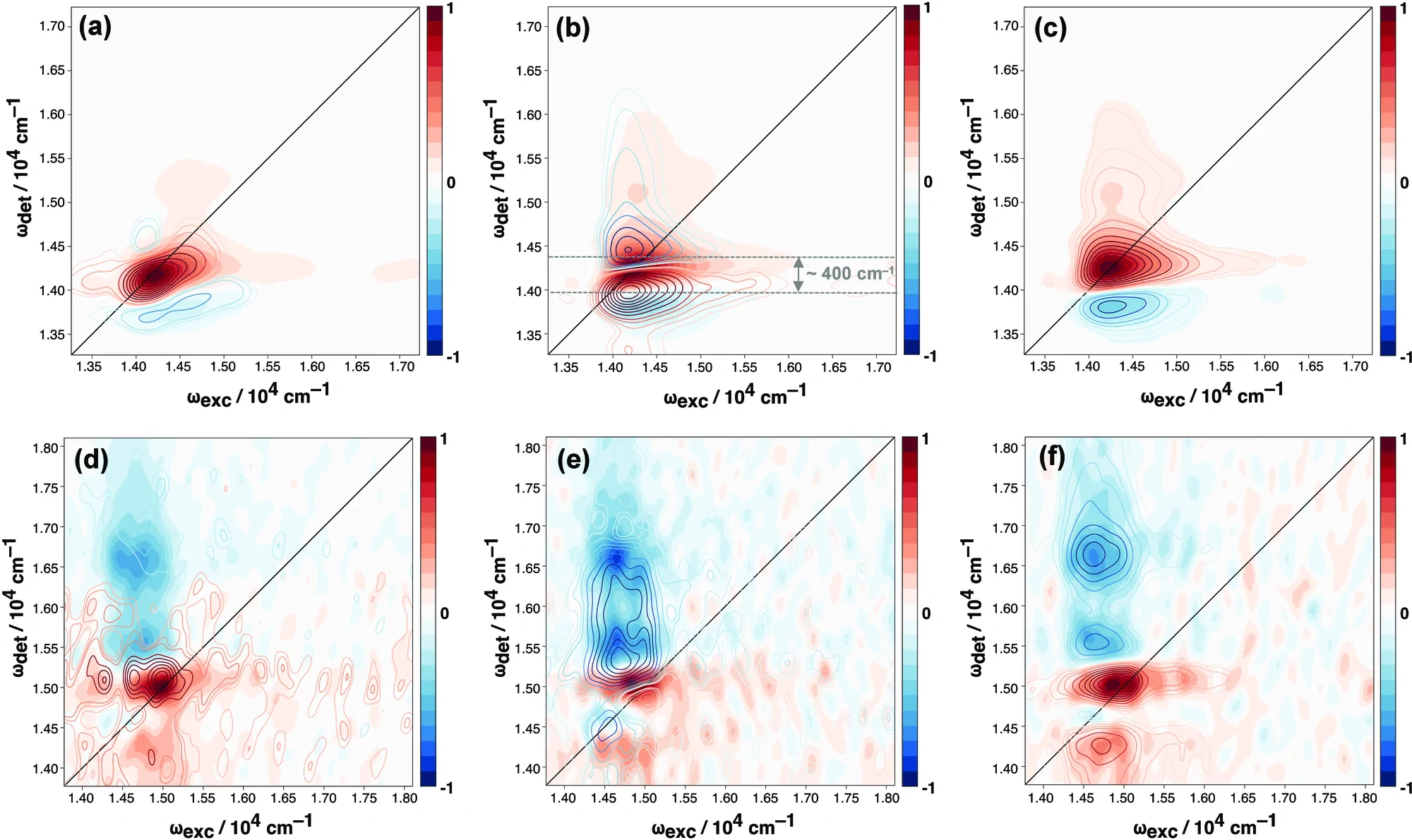
(a–c) 2D-DAS maps (contours) and corresponding absorptive 2DES (underlying area) associated with the 30 fs (a), 335 fs (b), and >1 ps (c) time constants for the J-aggregate under Set I excitation conditions. (d–f) 2D-DAS maps (contours) and corresponding absorptive 2DES (underlying area) associated with the 15 fs (d), 250 fs (e) and >1 ps (f) time constants for the J-aggregate under Set II excitation conditions. In all panels, the color scale is normalized to the 2DES map.

The longest time component (Figure 4c) depicts the complete restoration of ground-state population on a time scale much longer than the currently investigated experimental window.

The first kinetic constant (Figure 4a) reflects an initial ultrafast decay of all the main features appearing in the maps, and it can thus be attributed to an energy reorganization process. Sub-100 fs relaxation times recorded on the relaxation dynamics of analogous systems (aggregates of bacteriochlorophylls in chlorosomes)^70^ were attributed to fast energy redistribution within and between coherent domains of the aggregates, and this interpretation is also consistent with the present case.

The second time component (335 fs) presents a distinctive amplitude distribution (Figure 4b). Specifically, this component depicts (i) a rise (decay) of the high-energy (low-energy) side of the main diagonal peak at 14170 cm^–1^ and (ii) a concurrent rise of the red-shifted ESA signal (i.e., an increase in negative signal amplitude). Such a signal distribution is inconsistent with simple intraband energy relaxation processes, such as exciton–phonon scattering. In that case, one would expect a rising (decaying) signal at lower (higher) energies, opposite to the behavior observed here. Moreover, these relaxation processes typically occur on much faster time scales, generally on the order of only a few tens of femtoseconds.^39,43,71–73^ The alternative hypothesis of relaxation through a low-energy dark state is also not fully compatible with the frequency distribution and temporal evolution of the features observed in the 2D-DAS.^74,75^ Rather, these features are consistent with EEA. Although EEA has traditionally been regarded as an incoherent process limited by exciton diffusion, more recent studies have revealed the existence of coherent EEA mechanisms, in which the microscopic annihilation rate depends on the phase relationship between exciton wave functions, and thus on the J or H nature of the aggregate.^69,76–80^ Previous work on cyanine J-aggregates has shown that such coherent EEA processes occur on time scales of ∼100–350 fs and give rise to red-shifted ESA features together with blue-shifted GSB signals, owing to the generation of energetically “hot” one-exciton states.^77^ All of these signatures are present in the current data, supporting the assignment of the 335 fs component to coherent EEA. On the hundreds-of-femtoseconds time scale, exciton migration results in one exciton relaxing back to the ground state, while the second is promoted to a higher excited state, from which it rapidly relaxes to a hot one-exciton state. This process manifests itself as a growing ESA feature accompanied by an increasing GSB contribution at slightly blue-shifted energies (orange arrows in Figure 5).

**Figure 5:**
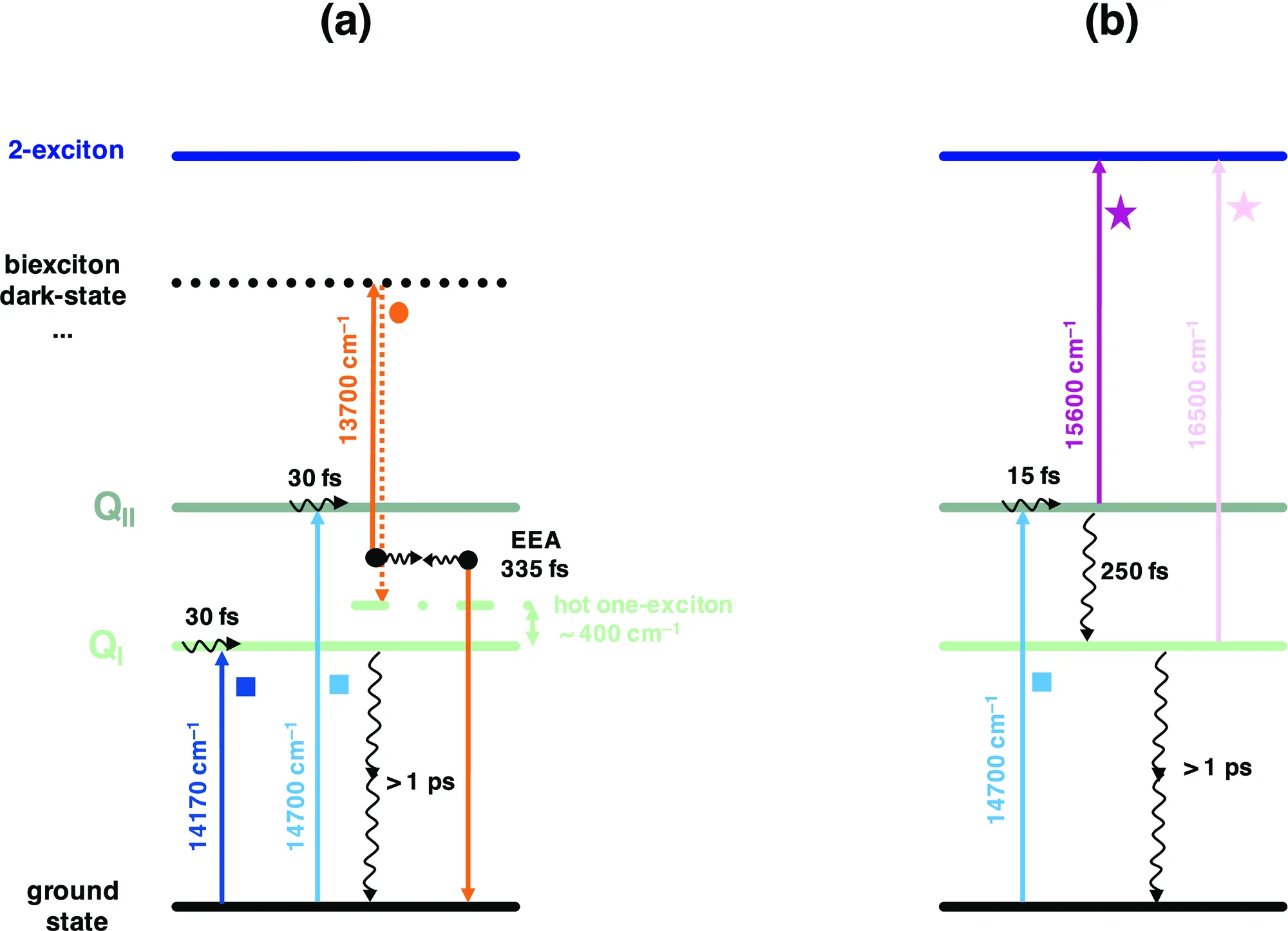
Schematic illustration of the energy-level structure of H_2_TPPS aggregates and of the dynamical processes captured by 2DES following photoexcitation of (a) both the Q_I_ and Q_II_ bands (Set I) and (b) the Q_II_ band only (Set II). Solid colored arrows denote the principal optical transitions contributing to the 2DES signals and are labeled with the corresponding transition frequencies, using the same color code and markers as in Figure 3. Wavy arrows indicate relaxation pathways and are labeled with the associated time constants obtained from the global multiexponential fitting.

Upon switching to Set II, which employs a blue-shifted excitation pulse profile, the 2DES maps exhibit a higher noise level, as expected given the significantly smaller transition dipole moments of the Q_II_ and Q_III_ bands compared to Q_I_. Under these conditions, the dominant feature in the maps is a positive diagonal peak at approximately (14965, 14965) cm^–1^, arising from GSB of the Q_II_ transition. Notably, although the Q_I_ transition lies at the lower boundary of the accessible spectral window, signatures of Q_*x*_–Q_*y*_ mixing remain detectable, as evidenced by the square pattern highlighted by the square markers in Figure 3b. In addition, two intense ESA features are observed from early population times at detection frequencies blue-shifted with respect to the main diagonal peak, at coordinates (14700, 15600) cm^–1^ (purple star) and (14700, 16500) cm^–1^ (pink star).

These signals are absent in set I and therefore emerge exclusively when the Q_II_ band is directly excited. Unlike the red-shifted ESA observed in set I, the spectral positions of these features are consistent with excited-state absorption from the one-exciton to the two-exciton manifold, in agreement with the Pauli exclusion principle, which dictates that the creation of a second exciton requires additional energy due to state-filling effects.^81^

Also for set II, the global multiexponential fitting analysis identified three kinetic constants: 15 fs, 250 fs, and a long component in the picosecond time scale.

The shortest and longest time constants agree with the first and third components previously identified in set I. Consistently, the corresponding 2D-DASs (Figure 4d,f, respectively) display amplitude distributions analogous to those observed in set I, allowing these components to be assigned to the same dynamical processes.

In contrast, the amplitude distribution of the 250 fs component (Figure 4e) exhibits a markedly different behavior. This suggests that despite the very similar numerical values of the second time constants in sets I and II, the corresponding component mainly originates from a distinct dynamical process. Specifically, the 2D-DAS at 250 fs shows decaying trends at the coordinates both of blue-shifted ESA features (pink and purple star markers) and of the main diagonal peak (light blue square marker), accompanied by a rising signal at the cross-peak below the diagonal (light green square marker).

As discussed above, the simultaneous decay of the signal at higher detection energies and the rise at lower detection energies is a characteristic signature of downward energy relaxation. Accordingly, the 250 fs component can be prevalently assigned to relaxation processes toward the bottom of the Q-band. Previous studies have reported similar time scales for internal conversion within the Q-band manifold, in good agreement with the present observations.

A comparison between the 2D-DASs in Figure 4e,f further reveals that the ESA feature at (14700, 15600) cm^–1^ (purple star) decays predominantly with the 250 fs component, whereas the higher-energy ESA at (14700, 16500) cm^–1^ (pink star) exhibits a stronger decay on the picosecond time scale. This behavior suggests that the former arises from instantaneous ESA involving higher-lying Q-band states, while the latter originates from transitions out of the lowest one-exciton states, which are progressively populated during the downward relaxation process, supporting our interpretation of the 250 fs process.

The comparative analysis between sets I and II permitted us to demonstrate that EEA is specifically prompted by the simultaneous excitation of Q_I_ and Q_II_ bands, while, when Q_I_ remains unexcited, this mechanism is significantly suppressed. In the schematic level structures of Figure 5, GSB/SE and ESA pathways originating from the two different laser profiles are summarized.

A possible explanation for the different dynamical behaviors observed under distinct excitation conditions can be found by considering the nature of the Q_I_ and Q_II_ transitions. As discussed in the context of the linear optical properties of the aggregates, it is now well established that the Q_I_ band originates predominantly from Q_*x*_ “J-type” molecular transitions, whereas the Q_II_ band is mainly characterized by Q_*y*_ “H-type” character.^33^

Tempelaar et al.^80^ theoretically demonstrated that the sign of the intermolecular dipole–dipole couplings has a profound impact on the EEA rate through interference effects arising from the phase relationships of the two-exciton wave functions. In H-type aggregates, this interference is destructive, leading to a strong suppression of the EEA rate. Subsequent experimental studies confirmed these predictions by comparing the dynamical responses of J- and H-type aggregates of perylene diimide^76^ and phthalocyanine derivatives.^78^

In the case of H_2_TPPS aggregates, the coexistence of J- and H-type subbands within the same system provides a unique opportunity to directly probe this interference mechanism by selectively exciting different bands. Specifically, excitation of the Q_I_ band (J-type, *x*-polarized) activates EEA, whereas selective excitation of the higher-energy Q_II_ band (H-type, *y*-polarized) limits EEA, resulting instead in more conventional downward energy relaxation dynamics.

Further information is provided by the beatings analysis in the two sets of measurements (Figures S6–S8 of the Supporting Information). In Set I, the analysis of the signal dynamics reveals the presence of oscillatory beatings in the signal amplitude, originating from coherent vibrational wave packets, as the extracted beating frequencies are in good agreement with those reported in resonant Raman spectra.^48^ The most significant beating modes have frequencies of 244 and 326 cm^–1^ (see Figure S6b of the Supporting Information) and exhibit dephasing times on the order of several hundred femtoseconds, as expected for nuclear degrees of freedom. The dominant mode at 244 cm^–1^ corresponds to the same out-of-plane ruffling motion previously observed in monomeric H_2_TPPS.

The intensity enhancement of the low-frequency Raman-active vibrations upon aggregate formation has already been reported,^66^ and the same modes have also been implicated in B-Q Herzberg–Teller coupling.^82^

These observations suggest that these low-frequency out-of-plane modes are strongly coupled to electronic transitions and play a significant role in modulating their properties.

While the 244 and 326 cm^–1^ modes strongly modulate the dynamical behavior of set I, no evidence of coherent oscillations is observed in set II. Although alternative explanations may partially account for this observation (including the poorer signal-to-noise ratio and a possible weaker coupling of the dominant vibrational modes to *y*-polarized transitions), the marked difference in the beating behavior between the two sets strongly supports the interpretation of the distinctive dynamics following selective Q_I_ excitation. Notably, these two low-frequency modes exhibit frequencies that approximately match the energy separation between the positive and negative lobes of the dispersive 2D-DAS associated with the 335 fs time constant (Figure 4b). This separation provides an estimate of the energy gap between the hot one-exciton state populated after the EEA event and the bottom of the Q-band. It is therefore reasonable to propose that, in the presence of EEA, the higher-energy state populated after annihilation relaxes toward lower-lying states, with the excess energy dissipated into specific phonon modes. This mechanism naturally explains the pronounced enhancement, observed in set I, of vibrational modes whose frequencies are in resonance with the relevant electronic energy gaps.

## 4 Conclusion

In conclusion, this work advances the extensive body of research on the optical properties and excited-state dynamics of the prototypical porphyrin derivative H_2_TPPS by investigating both its monomeric and its J-aggregated forms. Using 2DES, we provide a comprehensive characterization of the low-energy Q-band manifold, whose intricate spectral structure arises from the interplay of multiple electronic transitions and their vibronic progressions. The importance of this spectral region is underscored by the central role played by analogous Q-band transitions in biological light-harvesting pigments involved in photosynthesis, as well as in technological applications such as photovoltaics, optoelectronics, and photodynamic therapy.

The signal distribution in the 2DES maps reveals strong coupling between the two main Q transitions, which share a common ground state, in close analogy to observations previously reported for chlorophyll aggregates.^39,54^ These results support a picture in which the dominant structure of the Q-band region in H_2_TPPS aggregates does not arise solely from vibronic coupling but rather reflects a splitting into distinct excitonic subbands originating from different geometrical arrangements and interactions of the transition dipole moments of the monomeric units along the *x* and *y* axes (Figure 1a).^63^ This interpretation revises the conventional view of the Q-band absorption profile and emphasizes the role of collective electronic effects in shaping the aggregate optical response.

By selectively tuning the excitation frequency, we further demonstrate that different regions of the Q-band manifold activate fundamentally distinct dynamical mechanisms. Preferential excitation of the lowest-energy *x*-polarized J-type Q_I_ band provides compelling evidence for the activation of a coherent EEA channel, whereas selective excitation of the *y*-polarized H-type Q_II_ band significantly limits annihilation.

These distinct dynamical behaviors can be rationalized by considering the different polarization properties of the Q_I_ and Q_II_ transitions. Recent theoretical predictions have indeed shown that EEA can be coherently controlled in H- and J-aggregates, with the sign of the dipole–dipole coupling between chromophores determining whether phase relationships interfere destructively, as in H-aggregates, or constructively, as in J-aggregates, in the annihilation rate. In this framework, our experimental observations provide direct spectroscopic evidence of such excitation-selective control of EEA in a porphyrin aggregate system.

Together, these findings highlight the pronounced sensitivity of ultrafast dynamics in H_2_TPPS J-aggregates to the excitation conditions and reveal the coexistence of multiple, energy-dependent relaxation channels within the Q-band manifold. More broadly, this work provides mechanistic insights relevant to biomimetic light-harvesting systems and establishes guiding principles for the rational design of functional excitonic materials with tailored annihilation and energy-dissipation properties.

## Supplementary Material



## Data Availability

Data available on request from the authors.

## Notes

Additional experimental details, materials, and methods, including sample preparation, experimental 2DES setup, additional 2DES, 2D-DAS, and 2D-CAS data, and power spectra, (PDF)

## #

Dipartimento di Scienze Chimiche, Biologiche, Farmaceutiche ed Ambientali (ChiBioFarAm), Università degli Studi di Messina, Messina 98166, Italy
