# Supplementary material for: Selective Q‑Band Excitation Modulates Exciton–Exciton Annihilation in Tetra(p‑Sulfonatophenyl)porphyrin J‑Aggregates
Source: ACS Phys Chem Au. 2026 Jul 4;6(5):1192–202. doi: 10.1021/acsphyschemau.6c00064 (PMC13614047; doi:10.1021/acsphyschemau.6c00064)
Supplement: Supplementary file 1 [file pg6c00064_si_001.pdf]

SUPPLEMENTARY INFORMATION

Selective Q-Band Excitation Modulates Exciton–Exciton Annihilation in Tetra-(*p*-Sulphonato)-Phenyl-Porphyrin J-Aggregates

Maria Francesca Cherchi,[1] Teresa Paviglianiti,[1,#] Dario Baldon,[1] Federico Toffoletti,[1] Elisabetta Collini[1]*

[1]Department of Chemical Sciences, University of Padova, Padova 35131, Italy
[#] present address: Dipartimento di Scienze Chimiche, Biologiche, Farmaceutiche ed Ambientali (ChiBioFarAm), Università degli Studi di Messina, Messina 98166, Italy

* elisabetta.collini@unipd.it

Contents

S1. Samples preparation

S2. Experimental 2DES setup

S3. Additional 2DES data

  S3.1 Rephasing and non-rephasing data

  S3.2 Non-normalized 2D-DAS

  S3.3 Power spectra

  S3.4 2D-CAS

S1. Samples preparation

Sample solutions were prepared starting from an aqueous solution of tetra-(p-sulfonatophenyl)porphyrin (TPPS, PorphyChem) at neutral pH. Chloridric acid (0.1 M) was added to adjust the pH to 2.2.[1] For the 2DES experiments, the concentration was tuned to obtain an absorbance of approximately 0.3 at the excitation wavelength in 1 mm optical path length cuvettes. The formation of J-aggregates in solution was promoted by the addition of NaCl to achieve high ionic strength.[1]

S2. Experimental 2DES setup

The 2DES experiments were carried out with the setup described in details in ref.[2] A Ti:Sapphire Coherent® Libra laser system generated output pulses at 800 nm at a repetition rate of 3 kHz with a pulse energy of 0.8 mJ. The output was coupled into a non-collinear optical amplifier (NOPA) (Light Conversion® TOPAS White), which enabled tuning of the central wavelength across the 500-1100 nm spectral range. Pulse compression to approximately 10 fs at the sample position was achieved using a prism compressor in combination with a Fastlite Dazzler pulse shaper. The final pulse duration was determined by Transient Grating Frequency-Resolved Optical Gating (TG-FROG) measurements performed under identical experimental conditions on dimethyl sulfoxide (DMSO) (Figure S1). At the sample position, the pulse energy is reduced to ~7nJ per pulse using a broadband half-wave plate and a polarizer.

The 2DES apparatus was based on a passively phase-stabilized BOXCARS geometry. A custom two-dimensional diffraction grating divided the beam into four identical replicas positioned at the vertices of a square: three served as excitation pulses, while the fourth acted as the local oscillator (LO) for heterodyne detection. Temporal delays between pulses were controlled using pairs of CaF_2 wedges, with one wedge in each pair mounted on a motorized stage to finely adjust the optical path length, providing sub-femtosecond timing accuracy (temporal resolution ~0.07 fs).

For each measurement, the coherence time t_1 (delay between the first and second pulses), the population time t_2 (delay between the second and third pulses), and the rephasing time t_3 (delay between the third pulse and the signal emission) were defined. Both rephasing and non-rephasing signal components were recorded by modifying the pulse sequence, and their sum enabled reconstruction of purely absorptive 2D maps. The acquired dataset consisted of a 3D array describing the evolution of 2D frequency–frequency correlation spectra as a function of t_2. In each map, the excitation (ω_{exc}) and detection (ω_{det}) frequency axes were obtained by Fourier transformation along t_1 and t_3, respectively. The population time t_2 was scanned from 0 to 1000 fs with a step size of 7.5 fs. A rotating-frame approach was used in all measurements. Each experiment was repeated at least five times to check reproducibility, and the results were averaged to improve the signal-to-noise ratio.

All experiments were conducted at room temperature, employing two different excitation conditions. For the H_2TTPS monomer and the J-aggregate (Set II), a bandwidth centred at 15260 cm^{-1} (655 nm) with a pulse duration of 11 fs was employed (Figure S1). The coherence time t_1 was scanned from 0 to 78 fs in 1.3 fs

increments for the rephasing measurements, and from 0 to 65 fs in steps of 1.3 fs for the non-rephasing measurements. The pulse energy at the sample was 7.2 nJ per pulse.

For the J-aggregate (Set I), the excitation bandwidth was centred at 14535 cm^{-1} (688 nm) with a pulse duration of 12 fs. The coherence time t_1 was varied from 0 to 80 fs in steps of 2 fs for the rephasing experiments, and from 0 to 64 fs with the same step size for the non-rephasing experiments. In this case, the pulse energy at the sample was 8 nJ per pulse.

The data were analysed with the global fitting methodology described in ref.[3] The error on the values of the time constants have been estimated by repeated measurements to be in the order of ~10-15 %.

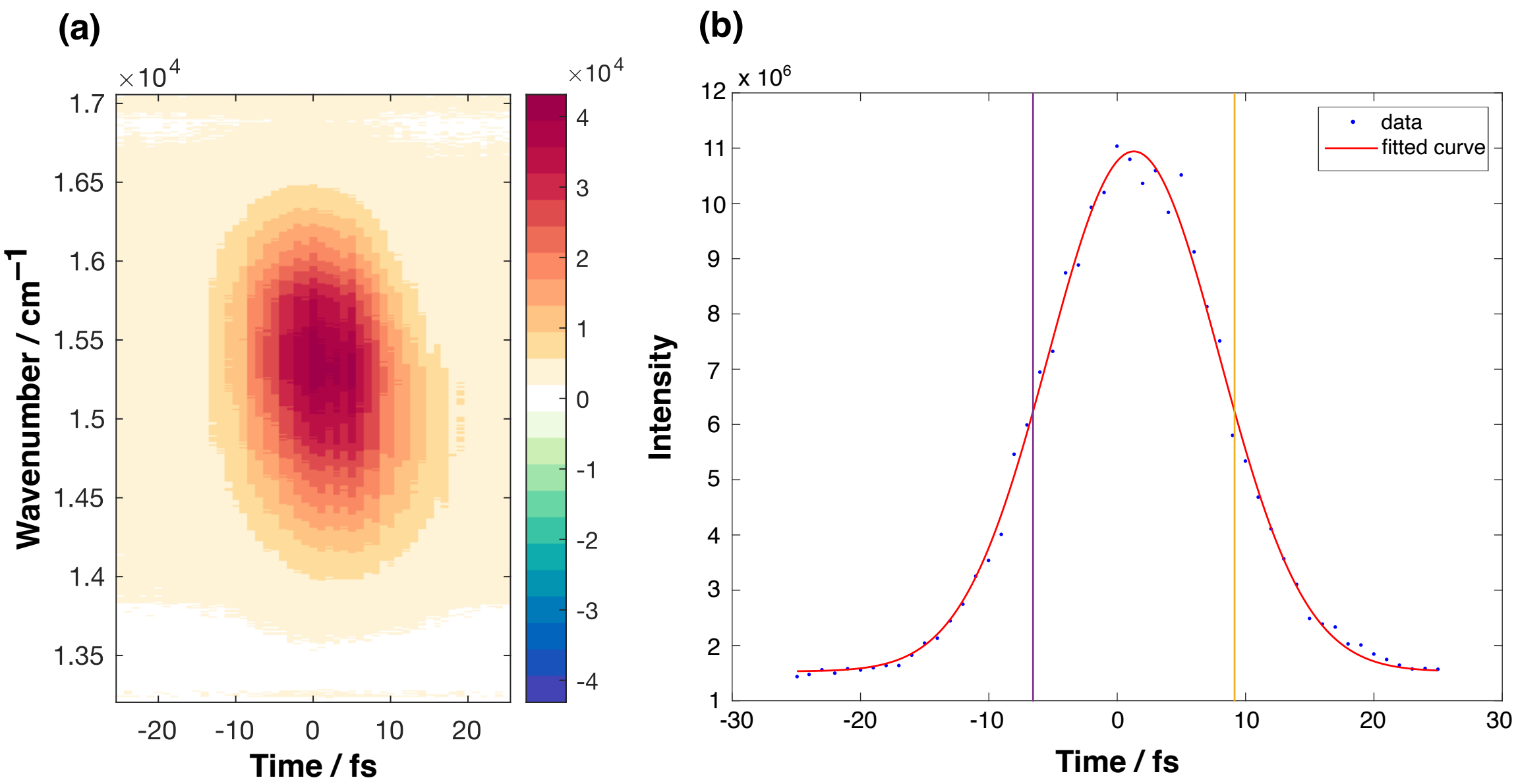

Figure S1. Pulse characterization by TG-FROG measurements (monomer and J-aggregate Set II). (a) Experimental pulse profile measured by the FROG experiment showing no relevant chirp effects. (b) Gaussian fit of the FROG signal integrated along the frequency dimension to estimate the pulse duration.

S3. Additional 2DES data

S3.1 Rephasing and non-rephasing data

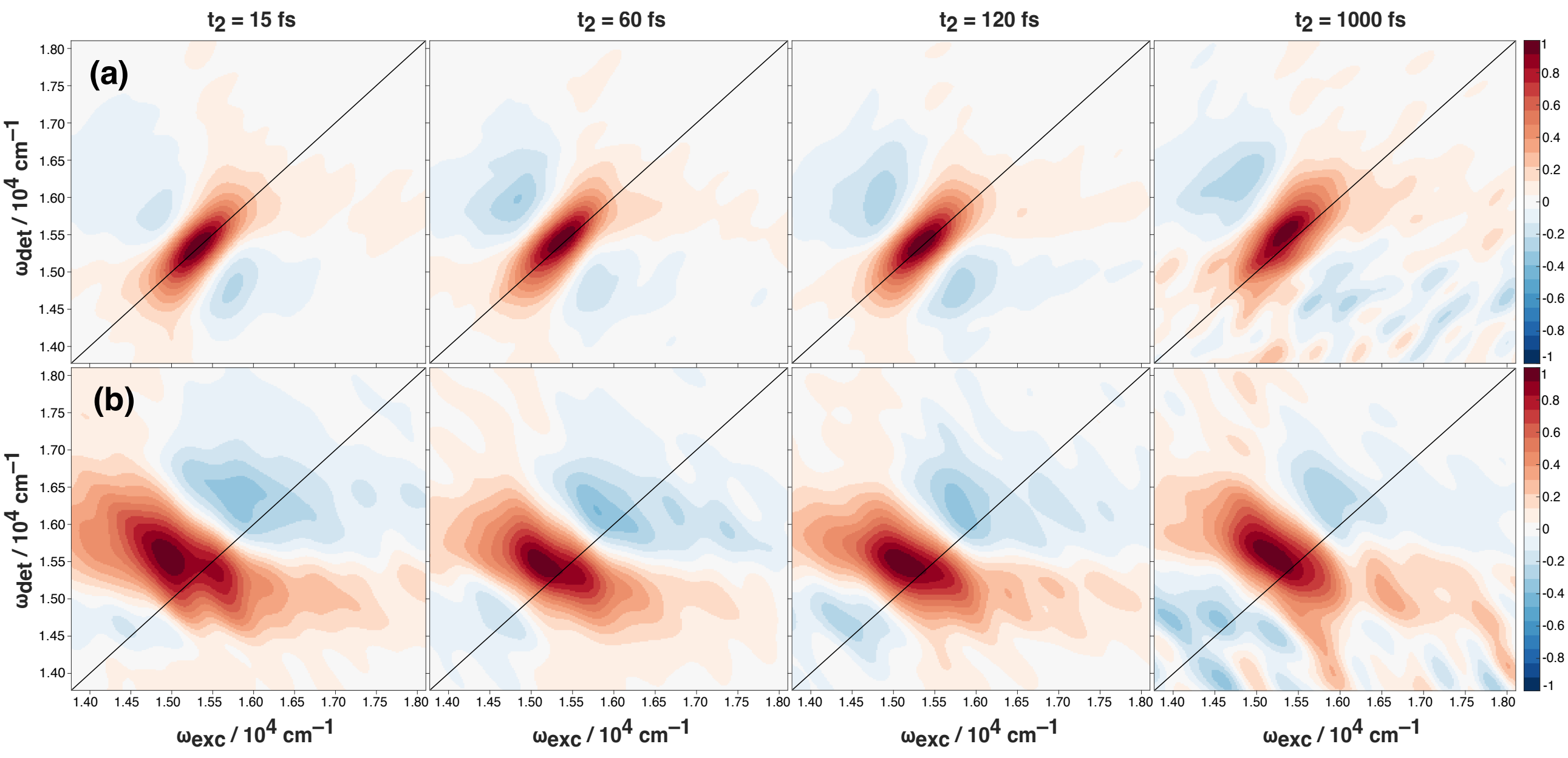

Figure S2. Real rephasing (a) and non-rephasing (b) 2DES spectra of the H_2TPPS monomer at selected values of t_2.

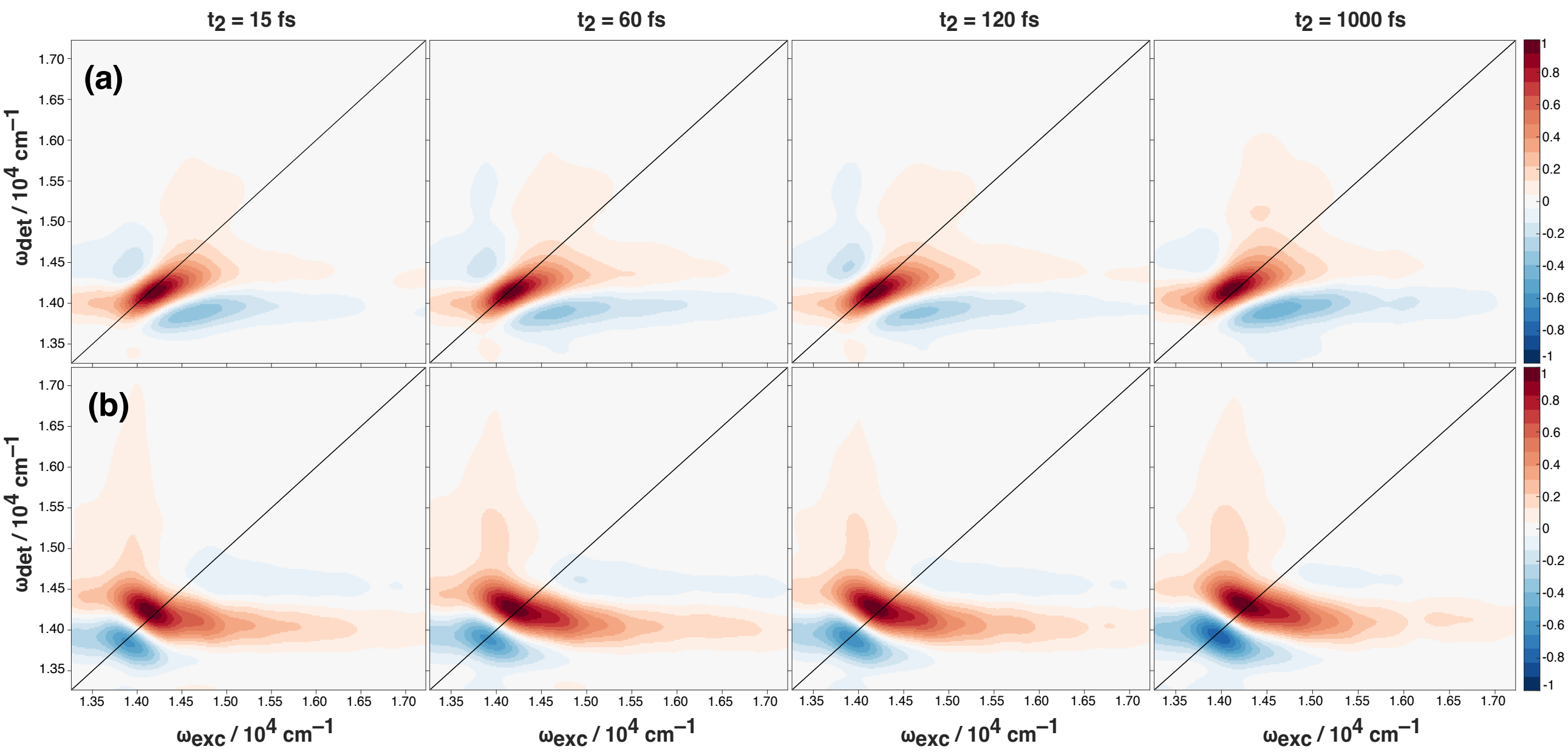

Figure S3. Real rephasing (a) and non-rephasing (b) 2DES spectra of the H_2TPPS J-aggregate (Set I) at selected values of t_2.

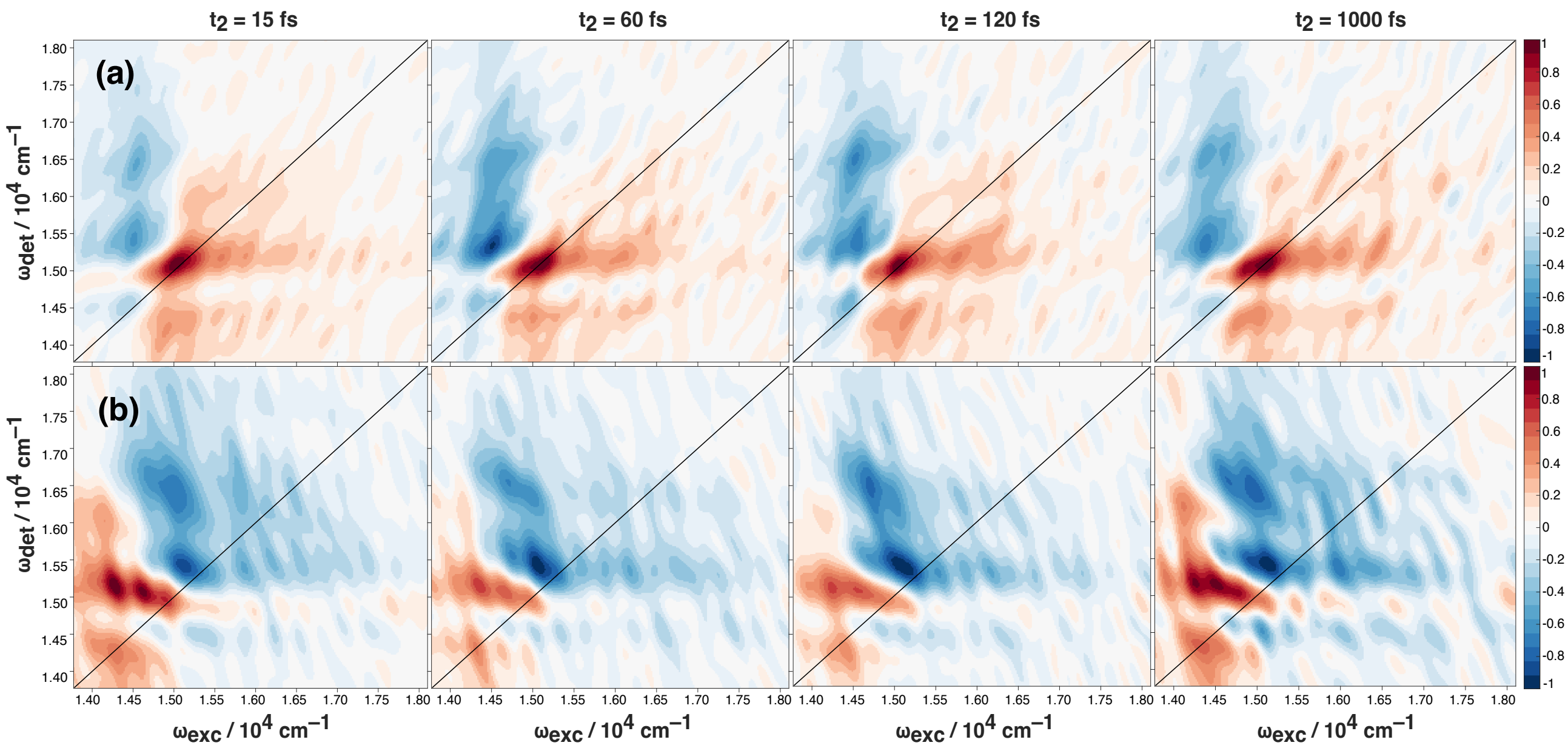

Figure S4. Real rephasing (a) and non-rephasing (b) 2DES spectra of the H_2TPPS J-aggregate (Set II) at selected values of t_2.

S3.2 Non-normalized 2D-DAS

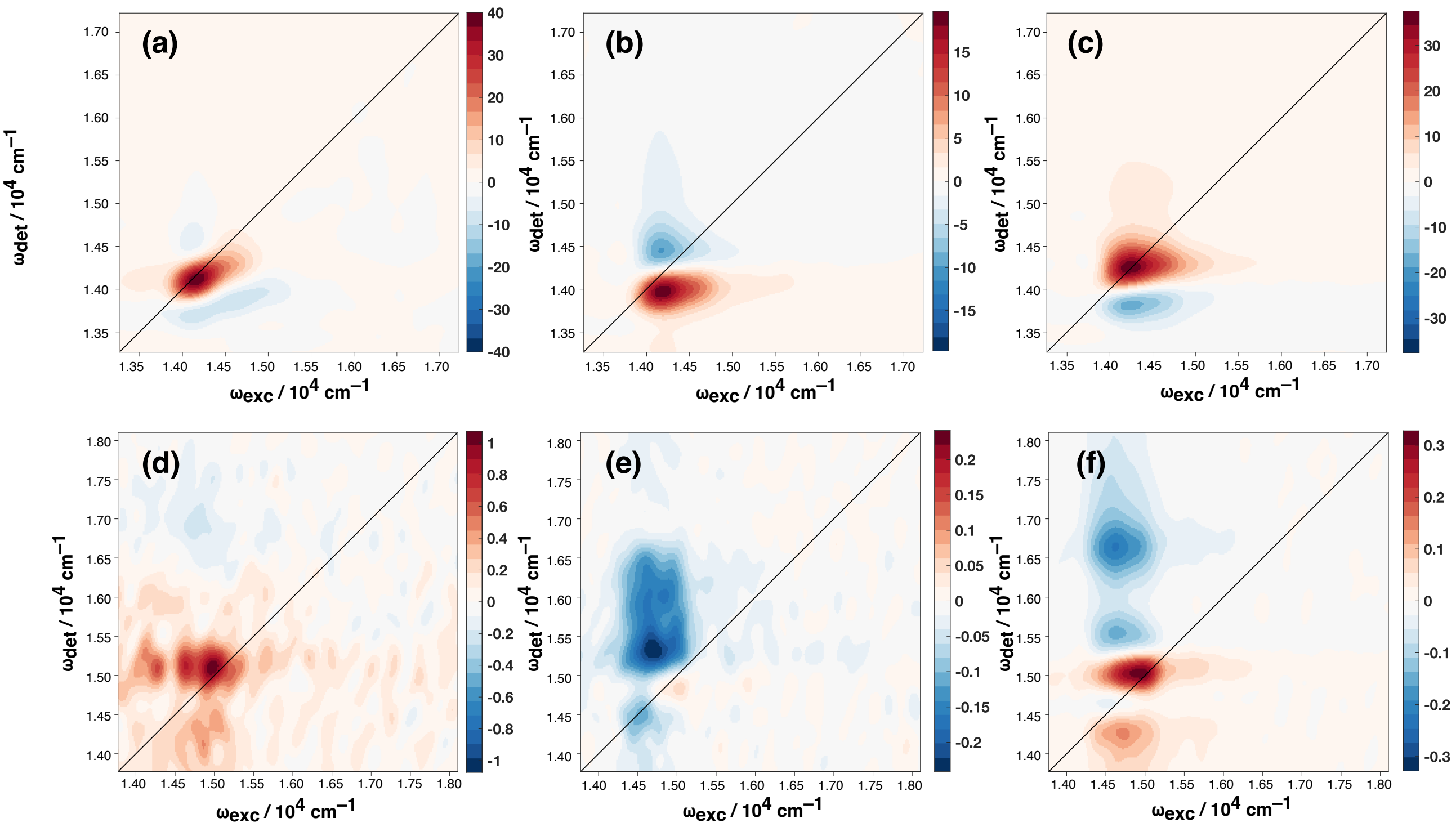

Figure S5. (a-c) Non-normalized 2D-DAS associated with the 30 fs (a), 335 fs (b), and > 1ps (c) time constants for the J-aggregate under Set I excitation conditions. (d-f) Non-normalized 2D-DAS maps associated with the 15 fs (d), 250 fs (e) and > 1ps (f) time constants for the J-aggregate under Set II excitation conditions.

S3.3 Power spectra

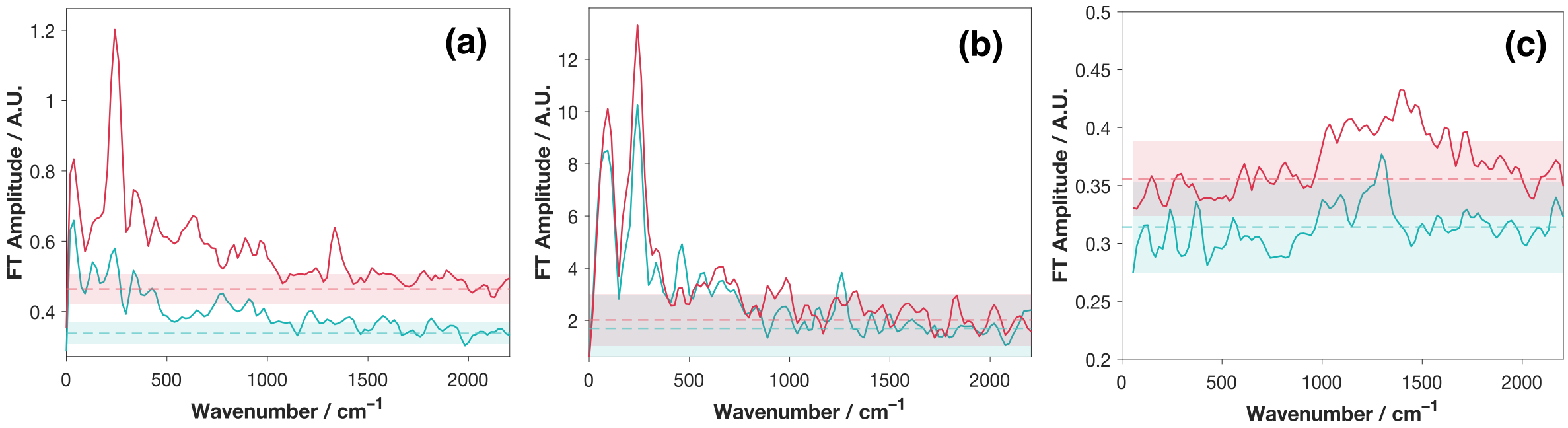

Figure S6. Power spectra of the rephasing (red) and non-rephasing (light blue) data for (a) H_2TPPS monomer, (b) H_2TPPS J-aggregate of Set I, and (c) H_2TPPS J-aggregate of Set II. A power spectrum is obtained as the squared magnitude of the Fourier transform of the oscillatory residuals after integration over both frequency dimensions.[4] The noise floor, highlighted by a dashed line, was estimated as the mean intensity in a signal-free spectral region (2000–2200 cm^{-1}); the shaded band represents the ±3σ detection threshold below which spectral features cannot be distinguished from background fluctuations.

S3.4 2D-CAS

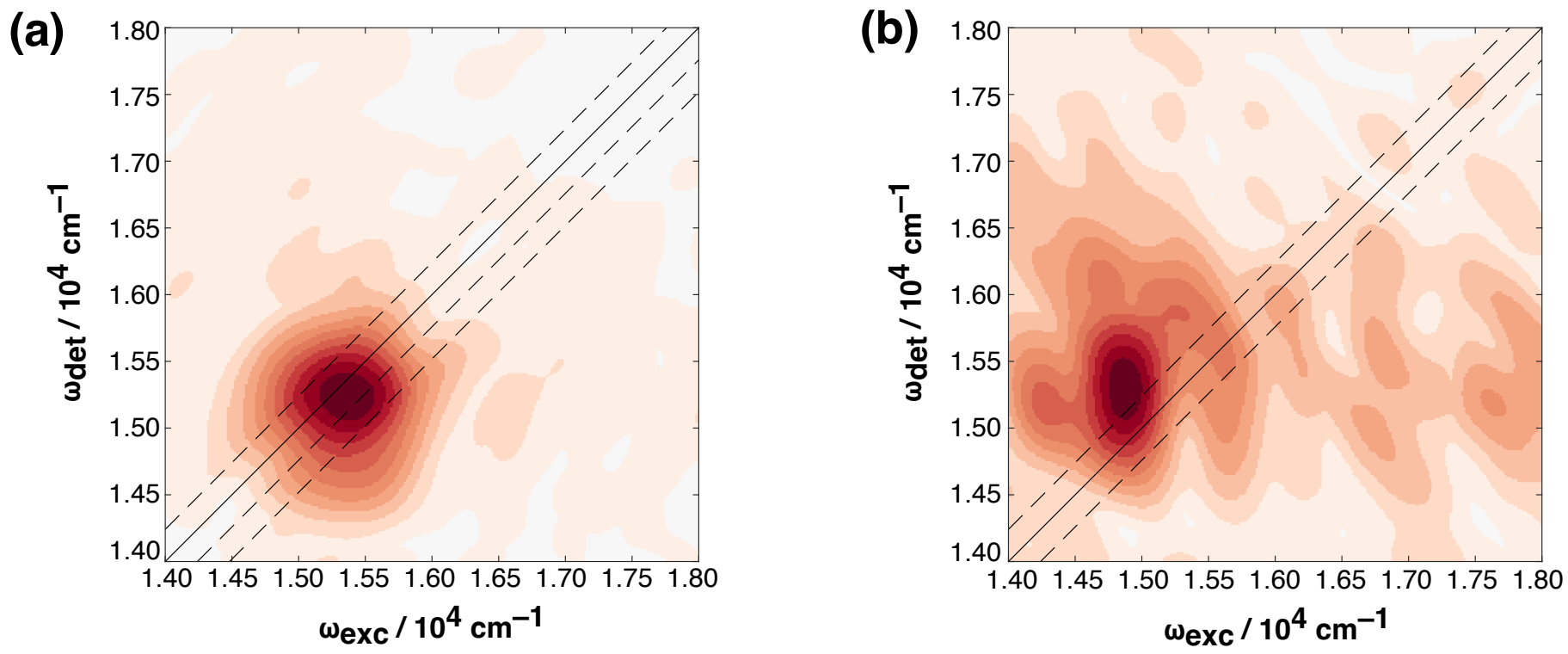

Figure S7. 2D-CAS relative to the main vibrational mode (ν_1=241 cm^{-1}, t_{deph} = ~ 500 fs) detected for H_2TPPS monomer in the rephasing (a) and non-rephasing (b) signal.

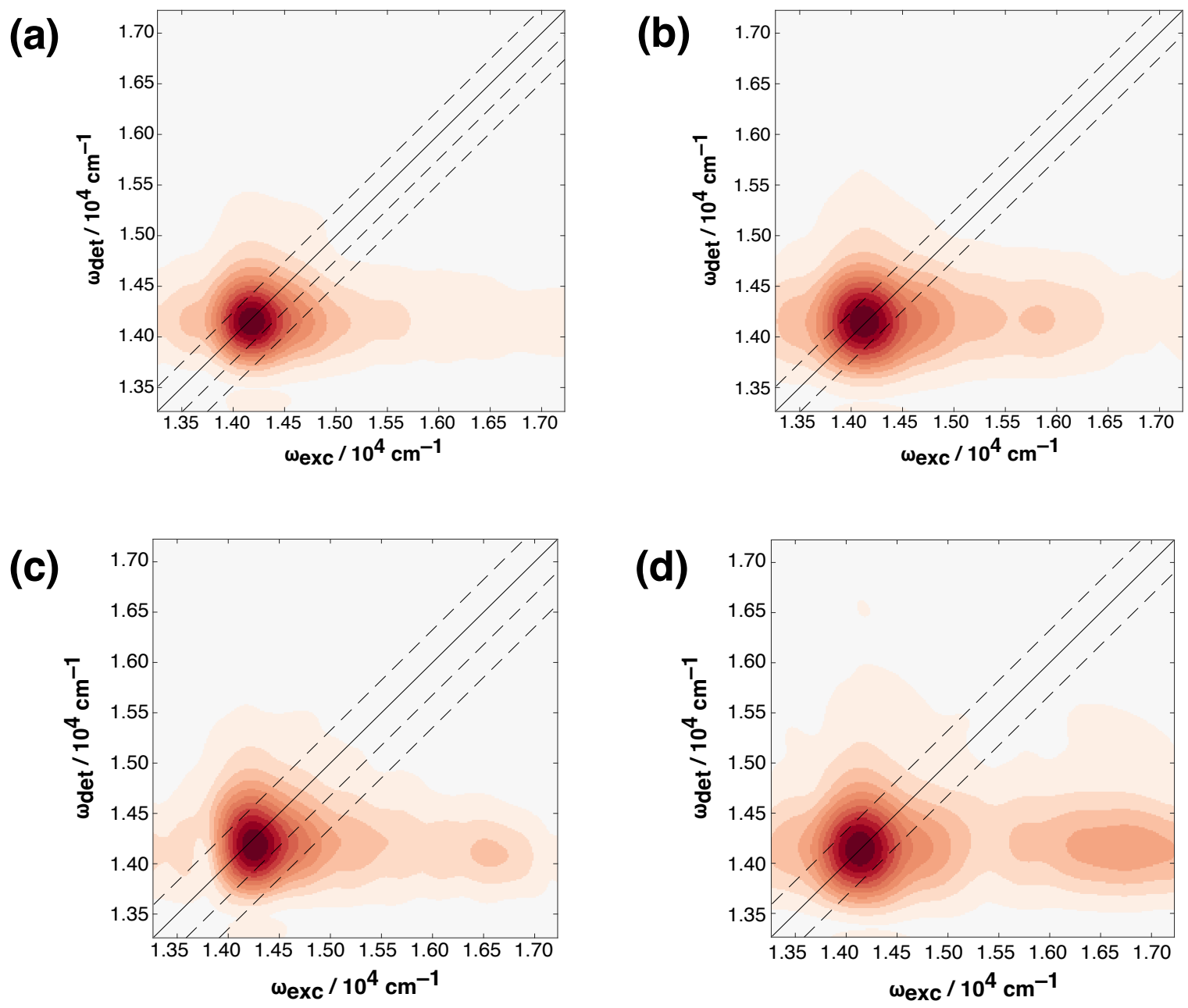

Figure S8. 2D-CAS of the oscillating components detected for the H_2TPPS J-aggregate (Set I). (a) Rephasing and (b) non-rephasing 2D-CAS of ν_1= 244 cm^{-1} (t_{deph} = 747 fs). (c) Rephasing and (d) non-rephasing 2D-CAS of ν_2= 326 cm^{-1} (t_{deph} > 1 ps).

References

(1) Micali, N.; Mallamace, F.; Romeo, A.; Purrello, R.; Monsù Scolaro, L. Mesoscopic Structure of Meso-Tetrakis(4-Sulfonatophenyl)Porphine J-Aggregates. *J. Phys. Chem. B* **2000**, *104* (25), 5897–5904. https://doi.org/10.1021/jp991909a.

(2) Bolzonello, L.; Volpato, A.; Meneghin, E.; Collini, E. Versatile Setup for High-Quality Rephasing, Non-Rephasing, and Double Quantum 2D Electronic Spectroscopy. *J. Opt. Soc. Am. B, JOSAB* **2017**, *34* (6), 1223–1233. https://doi.org/10.1364/JOSAB.34.001223.

(3) Volpato, A.; Bolzonello, L.; Meneghin, E.; Collini, E. Global Analysis of Coherence and Population Dynamics in 2D Electronic Spectroscopy. *Opt. Express, OE* **2016**, *24* (21), 24773–24785. https://doi.org/10.1364/OE.24.024773.

(4) Meneghin, E.; Pedron, D.; Collini, E. Raman and 2D Electronic Spectroscopies: A Fruitful Alliance for the Investigation of Ground and Excited State Vibrations in Chlorophyll *a*. *Chemical Physics* **2018**, *514*, 132–140. https://doi.org/10.1016/j.chemphys.2018.03.003.
